# Comprehensive analysis of coagulation indices for predicting survival in patients with biliary tract cancer

**DOI:** 10.1186/s12885-021-08684-w

**Published:** 2021-08-25

**Authors:** Xindi Ke, Bao Jin, Wen You, Yang Chen, Haifeng Xu, Haitao Zhao, Xin Lu, Xinting Sang, Shouxian Zhong, Huayu Yang, Yilei Mao, Shunda Du

**Affiliations:** 1 Department of Liver Surgery, Peking Union Medical College (PUMC) Hospital, PUMC & Chinese Academy of Medical Sciences, Beijing, 100730 China; 2 Peking Union Medical College (PUMC), Chinese Academy of Medical Sciences & PUMC, Beijing, China

**Keywords:** Biliary tract neoplasms, Coagulation, Prognosis, Nomogram

## Abstract

**Background:**

Abnormal activation of the coagulation system has been reported in patients with malignancies, but its prognostic significance in biliary tract cancer (BTC) remains unclear. This study aims to analyze and compare the prognostic value of coagulation indices in patients with BTC.

**Methods:**

The medical records of 450 patients with BTC who underwent surgical resection at our hospital between 2003 and 2017 were retrospectively analyzed. Time-dependent receiver operating characteristic curves were plotted to compare the predictive accuracy of coagulation indices. A predictive nomogram for overall survival (OS) was established based on the Cox regression analysis and validated in both the training and validation cohorts. A novel stratification model was created according to the total points of the nomogram.

**Results:**

Fibrinogen and international normalized ratio (INR) had the best predictive accuracy among the coagulation indices considered and were also the independent prognostic factors for OS. The nomogram and the novel stratification model had satisfactory performance and outperformed TNM staging.

**Conclusions:**

The study demonstrated that coagulation indices are valuable in predicting OS in BTC, with fibrinogen and INR having the best predictive ability. The nomogram and the novel stratification model could be applied to predict survival for patients with BTC.

**Supplementary Information:**

The online version contains supplementary material available at 10.1186/s12885-021-08684-w.

## Background

Biliary tract cancers (BTC), which comprise approximately 3% of gastrointestinal malignancies, are a relatively rare group of malignancy arising from the epithelia lining of the biliary tree. They are classified by anatomical origin into intrahepatic cholangiocarcinoma (ICC), extrahepatic cholangiocarcinoma (ECC), and gallbladder cancer (GBC). The incidence of BTC is relatively low in the Western world, ranging from 0.35 to 2 per 100,000 annually; however, this rate is higher in the Andes region and some areas of Asia [1]. Currently, the occurrence of GBC is on the decline in the western world [1], the incidence of cholangiocarcinoma displays an increasing trend worldwide [2]. BTC is characterized by a dismal prognosis and surgical resection is the only potentially curative option for all BTC subtypes [3, 4]. The prognosis of BTC after surgery, however, is still far from optimistic due to high rates of recurrence and metastasis [5, 6]. The survival prediction of patients with BTC after operation is currently reliant on surgical and pathological factors. Thus, there is an urgent need to investigate the patterns of BTC patients’ survival in order to determine other potent prognostic factors.

Since Trousseau discovered the tendency for thrombotic disorders to occur in cancer patients in 1865, the relationship between cancer and the coagulation pathway has been increasingly investigated [7, 8]. Aberrant activation of the coagulation pathway is frequently observed in patients suffering from cancer, typically manifesting as subclinical abnormalities in conventional blood coagulation tests [9]. Diverse mechanisms have been proposed for the coagulopathy associated with cancer, primarily including the expression of procoagulant proteins by tumor cells, the production of inflammatory cytokines, and the promoted adhesion of tumor cells to host cells [10]. The tumor-associated disturbance of the coagulation system contributes to tumor growth, progression, and metastasis, worsening the prognosis of patients with cancer [11]. In addition, the procoagulant state tends to result in venous thromboembolism, disseminated intravascular coagulation, and hemorrhage, which are commonly observed and potentially life-threatening complications for patients with cancer [11, 12]. Thus, it is reasonable that coagulation indices have potential prognostic value in malignant neoplasms. What’s more, the clinical significance of international normalized ratio (INR) has been acknowledged in hepatocellular carcinoma, as INR is one of the essential factors for the Child–Turcotte–Pugh score, which has been integrated in the Barcelona Clinic Liver Cancer staging system. Therefore, coagulation indices from routine coagulation function tests are likely valuable in the prediction of survival in BTC. Although a few studies have already reported the prognostic significance of coagulation assays in some malignancies [13–18], research regarding the relationship between coagulation indices and the prognosis of BTC is still limited.

This study aimed to elucidate and compare the prognostic value of preoperative coagulation indices, including fibrinogen (FBG), prothrombin time (PT), prothrombin time activity (PTA), INR, activated partial thromboplastin time (APTT), activated partial thromboplastin time ratio (APTT-R), thrombin time (TT), and platelet count (PLT), in patients with BTC who underwent surgical resection. In addition, we sought to establish and validate a nomogram integrating independent prognostic factors for OS prediction and construct a novel stratification model for BTC patients.

## Methods

### Patients

The medical records of all patients with BTC at Peking Union Medical College Hospital between January 2003 and December 2017 were retrospectively reviewed. Inclusion criteria was established: 1) pathologically proven ICC, ECC, or GBC; 2) no history of other malignant tumors; 3) underwent surgical resection of BTC; and 4) complete measurement of coagulation indices, including FBG, PT, PTA, INR, APTT, APTT-R, TT, and PLT. Exclusion criteria was established: 1) incomplete follow-up data; 2) history of previous anti-tumor treatment before operation; 3) thromboembolic events or active infections before surgery; and 4) perioperative mortality or postsurgical survival of less than 1 month. In total, 450 eligible patients were enrolled for analysis in this study. The entire cohort was divided randomly into the training (*n* = 300) and validation (*n* = 150) cohorts at a ratio of 2:1.

### Data collection

The medical documents of patients with BTC at our hospital were collected for demographic and clinicopathologic data, including age, sex, comorbidities, tumor differentiation, tumor size, tumor-node-metastasis (TNM) stage, tumor markers, liver function tests, coagulation indices, routine blood tests, details of operation, and postsurgical complications. Fasting venous blood samples in the early morning were obtained within 5 days preoperatively in order to collect preoperative hematological parameters. The patients were staged by experienced clinical practitioners according to the 7th edition of the American Joint Committee on Cancer (AJCC) TNM staging system for ICC, ECC, or GBC, respectively. Curative surgery was defined as complete resection of primary and invasive lesions with R0 margin status, and lymphadenectomy if lymph node metastases were present. Although patients classified as stage IV are not theoretically eligible for surgical resection, these patients were not excluded from this study since they underwent disease progression during the waiting period for surgery or their TNM stage could not be assessed until metastases were found during exploratory laparotomy. For these patients, non-curative surgery was performed.

The patients were required to return to our hospital every 3 months in the first 2 years after the operation and every 6 months in the third year. Thereafter, patients with no signs of recurrence returned annually for follow-up. Telephone calls were conducted for follow-ups if patients did not visit our hospital on schedule. The endpoint of this study was OS, which was defined as the interval between the date of surgery and the date of death or the last follow-up.

### Statistical analysis

The optimal cut-off values of the coagulation indices were determined using X-tile software, while clinical references were used as the cut-off values for carbohydrate antigen 19-9 (CA19-9) and liver function parameters, such as alanine aminotransferase (ALT), aspartate aminotransferase (AST), albumin (ALB), and γ-glutamyl transpeptidase (GGT). Continuous variables were presented as medians with ranges, while numbers and percentages were reported for categorical variables. Comparisons of continuous variables were performed with the Wilcoxon rank-sum test as they were all abnormally distributed, as revealed by the Kolmogorov–Smirnov test and P-P plots. Categorical variables between groups were compare by the Pearson χ^*2*^ test or Fisher’s exact test, as appropriate. Specially, TNM stage was regarded as ordinal categorical variable; thus, the Wilcoxon rank-sum test was used to determine whether there was any significant difference.

Kaplan-Meier survival curves were plotted to depict the distributions of OS and significant differences were determined by the log-rank test. Time-dependent area under receiver operating characteristic (AUC) curves were generated to compare the prognostic value of these coagulation indices. Cox regression analyses were performed in the training cohort to select independent risk factors. All statistically significant variables in the univariate analysis were included in the multivariate analysis. A predictive nomogram integrating all independent prognostic factors associated with OS was established. The performance of the nomogram was evaluated by Harrell’s concordance index (C index), calibration curve, and decisive curve analysis (DCA) in both the training and validation cohorts. The nomogram was also validated in ICC, ECC, and GBC, respectively. A novel stratification model, which classified the patients into low-risk, middle-risk, and high-risk groups, was constructed using X-tile software according to the total points based on the nomogram. The novel stratification model was compared with TNM stage of the AJCC 7th edition by Kaplan-Meier survival curves and the C index.

Statistical analyses were performed with SPSS 26.0 (IBM Corporation, Armonk, NY, USA) and R software version 3.6.2 (http://www.r-project.org/). For all tests and analyses, a *P*-value less than 0.05 was considered statistically significant.

## Results

### Baseline characteristics

A total of 450 eligible patients with BTC were included in the study and their data were retrospectively analyzed. There were 99 (22.0%), 225 (50.0%), and 126 (28.0%) patients diagnosed with ICC, ECC, and GBC, respectively. The follow-ups were censored in February 2020 and 289 patients were confirmed dead by the last follow-up. The median OS was 23 months with 1-, 3-, and 5-year OS rates of 67.7, 39.6, and 28.6%, respectively. Baseline characteristics of all participants are summarized in Table 1. Among the participants, 208 (46.2%) were female and 257 (57.5%) underwent curative surgery. The patients were 23–89 years old with the median age of 62 years. There were 141 (31.3%), 131 (29.1%), 127 (28.2%), and 51 (11.4%) patients classified into TNM stage I, II, III, and IV, respectively.

**Table 1:** Baseline characteristics

Characteristics	Entire cohort	Training cohort	Validation cohort	*P*
Age (year)	62 (23–89)	62 (35–89)	63 (23–88)	0.120
Sex				0.947
Female	208 (46.2%)	139 (46.3%)	69 (46.0%)	
Male	242 (53.8%)	161 (53.7%)	81 (54.0%)	
Malignancy type				0.984
ICC	99 (22.0%)	65 (21.7%)	34 (22.7%)	
ECC	225 (50.0%)	152 (50.7%)	73 (48.7%)	
GBC	126 (28.0%)	83 (27.6%)	43 (28.6%)	
Tumor differentiation	[425]	[286]	[139]	0.521
Poor	162 (38.1%)	106 (37.1%)	56 (40.3%)	
Modest-well	263 (61.9%)	180 (62.9%)	83 (59.7%)	
Tumor size (cm)	2.5 (0.2–18.0)	2.5 (0.2–18.0)	2.2 (2.0—13.0)	0.751
TNM stage				0.619
I	141 (31.3%)	100 (33.3%)	41 (27.3%)	
II	131 (29.1%)	84 (28.0%)	47 (31.3%)	
III	127 (28.2%)	82 (27.3%)	45 (30.00%)	
IV	51 (11.4%)	34 (11.4%)	17 (11.4%)	
Curative surgery	[447]	[297]		0.511
No	190 (42.5%)	123 (41.4%)	67 (44.7%)	
Yes	257 (57.5%)	174 (58.6%)	83 (55.3%)	
Jaundice				0.423
No	210 (46.7%)	136 (45.3%)	74 (49.3%)	
Yes	240 (53.3%)	164 (54.7%)	76 (50.7%)	
Diabetes				0.239
No	362 (84.4%)	246 (82.0%)	116 (77.3%)	
Yes	88 (19.6%)	54 (18.0%)	34 (22.7%)	
Hypertension				0.070
No	314 (69.8%)	201 (67.0%)	113 (75.3%)	
Yes	136 (30.2%)	99 (33.0%)	37 (24.7%)	
Fatty liver				0.168
No	422 (93.8%)	278 (92.7%)	144 (96.0%)	
Yes	28 (6.2%)	22 (7.3%)	6 (4.0%)	
Liver cirrhosis				0.265
No	435 (96.7%)	288 (96.0%)	147 (98.0%)	
Yes	15 (3.3%)	12 (4.0%)	3 (2.0%)	
FBG (g/L)	3.765 (1.53–13.00)	3.74 (1.53–13.00)	3.77 (1.61–12.50)	0.566
PT (s)	11.7 (2.4–18.3)	11.7 (2.4–18.3)	11.7 (9.7–18.2)	0.288
PTA (%)	91.25 (43.5–126.4)	91.7 (43.5–126.4)	90.4 (49.8–116.6)	0.220
INR	1.00 (0.75–3.71)	0.995 (0.75–3.71)	1.01 (0.80–1.92)	0.148
APTT (s)	26.4 (17.2–51.3)	26.45 (17.2–51.3)	26.35 (19.1–48.1)	0.957
APTT-R	0.98 (0.63–2.75)	0.98 (0.63–2.75)	0.975 (0.71–1.77)	0.816
TT (s)	18.0 (10.2–24.4)	18.1 (10.2–24.4)	17.8 (14.2–22.5)	0.136
PLT (×10^9^/L)	227 (76–773)	228.5 (92–773)	224 (76–506)	0.736
CA19–9 (U/mL)	[438]	[290]	[148]	0.840
≤ 37	124 (28.3%)	83 (28.6%)	41 (27.7%)	
> 37	314 (71.7%)	207 (71.4%)	107 (72.3%)	
ALT (U/L)				0.539
≤ 40	177 (39.3%)	121 (40.3%)	56 (37.3%)	
> 40	273 (60.7%)	179 (59.7%)	94 (62.7%)	
AST (U/L)	[429]	[290]	[139]	0.192
≤ 40	186 (43.4%)	132 (45.5%)	54 (38.8%)	
> 40	243 (56.6%)	158 (54.5%)	85 (61.2%)	
GGT (U/L)	[432]	[293]	[139]	0.872
≤ 40	82 (19.0%)	55 (18.8%)	27 (19.4%)	
> 40	350 (81.0%)	238 (81.2%)	112 (80.6%)	
ALB (g/L)	[448]	[299]	[149]	0.725
≤ 35	89 (19.9%)	58 (19.4%)	31 (20.8%)	
> 35	359 (80.1%)	241 (80.6%)	118 (79.2%)	
Blood loss during surgery (mL)	300 (0–8000)	300 (0–8000)	300 (0–4000)	0.820
Postoperative hospitalization (day)	14 (1–155)	14 (1–155)	12 (2–91)	0.294
Postoperative complication				0.447
No	284	193	91	
Yes	166	107	59	

*P*-values in the last column belong to the training and validation cohorts. Asterisks indicate *P*-values of statistical significance. Continuous variables were shown as medians and ranges, and categorical variables were reported as numbers and percentages. For variables with missing data, the numbers of available cases were shown in square brackets

The entire cohort was randomly split into the training (*n* = 300) and validation (*n* = 150) cohorts. Baseline characteristics of the training and validation cohorts are also shown in Table 1. There was no significant difference in any clinicopathological characteristics between the two cohorts. At last, 287 participants in the training cohort were included in the development of the nomogram after excluding 13 patients with missing data for CA19–9 or curative surgery. Similarly, 148 participants without any missing data in the validation cohort were available to validate the nomogram established in the training cohort.

### Comparison of the prognostic value of coagulation indices

**Fig. 1:**
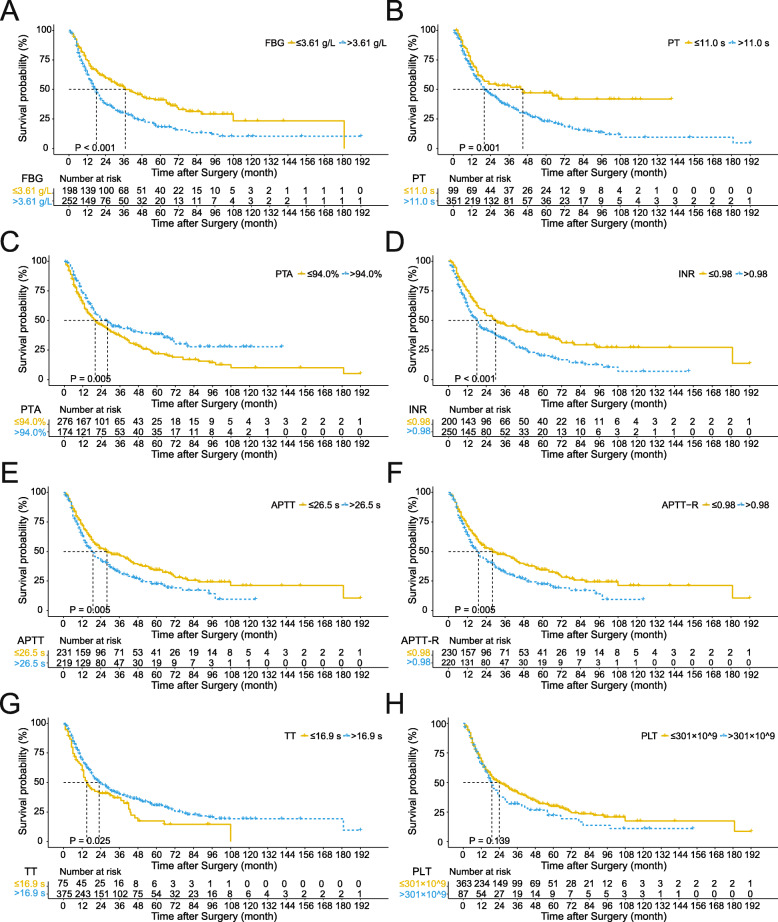
Kaplan-Meier survival curves for overall survival stratified according to coagulation indices. **a** FBG ≤ 3.61 g/L vs FBG > 3.61 g/L. **b** PT ≤ 11.0 s vs PT > 11.0 s. **c** PTA ≤ 94.0% vs PTA > 94.0%. **d** INR ≤ 0.98 vs INR > 0.98. **e** APTT≤26.5 s vs APTT> 26.5 s. **f** APTT-R ≤ 0.98 vs APTT-R > 0.98. **g** TT ≤ 16.9 s vs TT > 16.9 s. **h** PLT ≤ 301 × 10^9^/L vs PLT > 301 × 10^9^/L

**Fig. 2:**
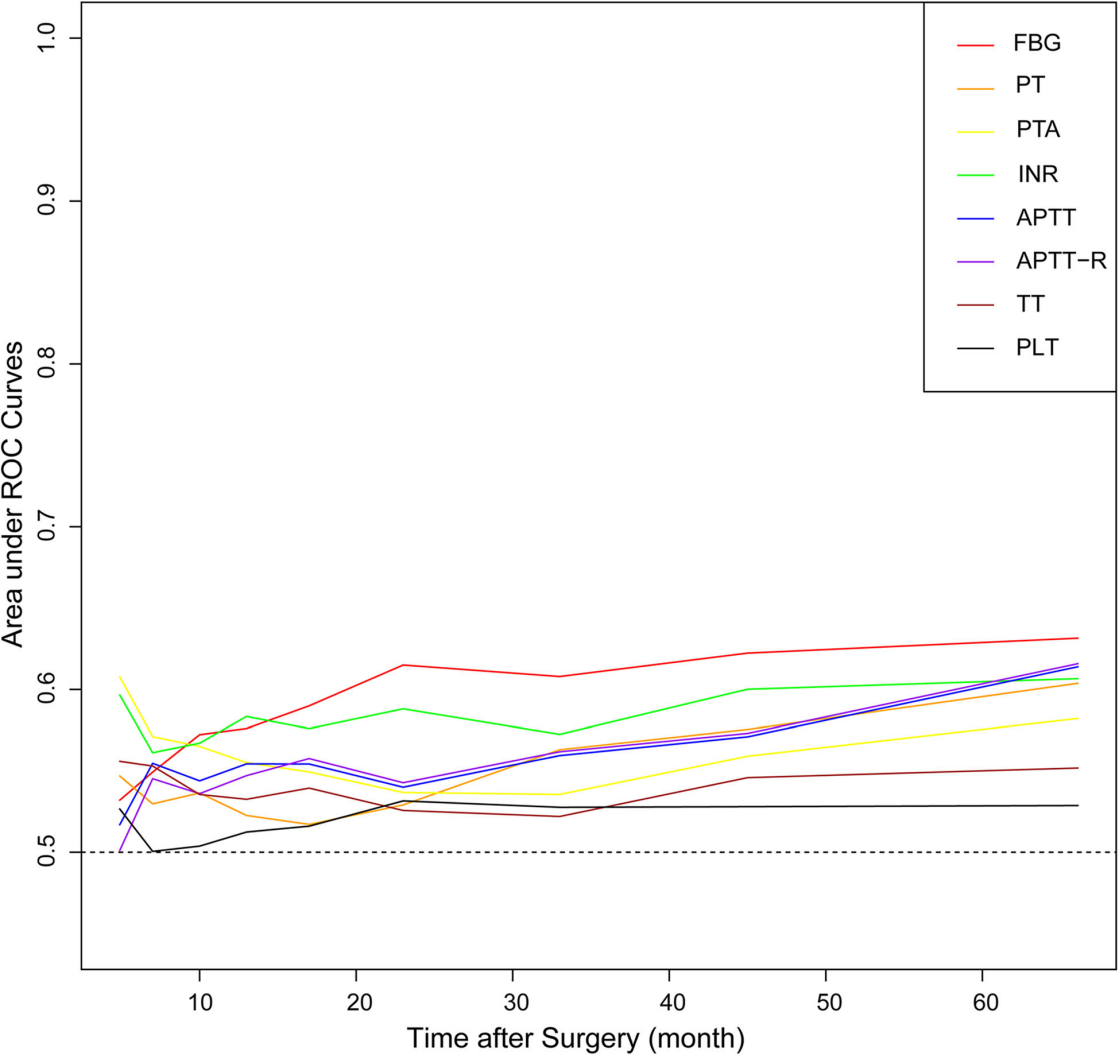
Time-dependent receiver operating characteristic curves of coagulation indices for survival prediction in patients with biliary tract cancer

Patients were stratified into groups according to the optimal cut-off values of FBG, PT, PTA, INR, APTT, APTT-R, TT, and PLT, which were 3.61 g/L, 11.0 s, 94.0%, 0.98, 26.5 s, 0.98, 16.9 s, and 301 × 10^9^/L, respectively. Patients with a preoperative level of FBG higher than 3.61 g/L had poorer OS than those with a low FBG (18 vs 37 months, *P* < 0.001, Fig. 1a). The median survival time of patients with a PT > 11.0 s was 20 months, which was significantly shorter than that of patients with a PT ≤ 11.0 s (45 months, *P* = 0.001, Fig. 1b). Likewise, dismal prognoses were also indicated by low PTA (20 vs 28 months, *P* = 0.005, Fig. 1c), high INR (18 vs 30 months, *P* < 0.001, Fig. 1d), prolonged APTT (19 vs 28 months, *P* = 0.005, Fig. 1e), high APTT-R (19 vs 28 months, *P* = 0.005, Fig. 1f), and shortened TT (15 vs 23 months, *P* = 0.025, Fig. 1g). Nonetheless, the median OS had no significant difference between the high PLT and low PLT groups (19 vs 24 months, *P* = 0.139, Fig. 1h). Time-dependent AUC curves showed that FBG ranked first and INR was superior to all indices except FBG over a wide range of time (Fig. 2), suggesting that FBG and INR had better predictive abilities than the other coagulation indices.

### Independent prognostic factors for OS

Cox regression analyses were conducted in the training cohort to select independent prognostic factors for OS (Table 2). Univariate analysis revealed that FBG (HR = 1.918, *P* < 0.001), PT (HR = 1.587, *P* = 0.013), INR (HR = 1.585, *P* = 0.001), APTT (HR = 1.382, *P* = 0.025), and APTT-R (HR = 1.380, *P* = 0.025) were adverse prognostic factors for OS, while PTA (HR = 0.718, *P* = 0.025) was a favorable prognostic factor. Only FBG (HR = 1.478, *P* = 0.023) and INR (HR = 1.879, *P* = 0.013), however, remained independent prognostic factors in the multivariate analysis. Advanced TNM staging (stage I as the reference, HR = 1.709 for stage II, HR = 1.727 for stage III, and HR = 2.637 for stage IV, all *P* < 0.05), curative surgery (HR = 0.438, *P* < 0.001), and elevated CA19-9 (HR = 1.644, *P* = 0.018) were also independently associated with OS.

**Table 2:** Cox Univariate and Multivariate Analysis

Variates	Cases (*n* = 300)	Univariate analysis		Multivariate analysis	
		HR (95% CI)	***P***	HR (95% CI)	***P***
Age (year)					
≤ 60	142	Reference			
> 60	158	1.190 (0.898–1.576)	0.225		
Sex					
Female	139	Reference			
Male	161	0.926 (0.699–1.226)	0.591		
Tumor Differentiation	[286]				
Poor	106	Reference		Reference	
Modest-well	180	0.656 (0.490–0.878)	0.005 *	0.766 (0.555–1.057)	0.104
Tumor size (cm)	[282]				
≤ 2	126	Reference		Reference	
> 2	156	1.379 (1.028–1.851)	0.032 *	1.066 (0.766–1.484)	0.703
TNM stage			< 0.001		0.001
I	100	Reference		Reference	
II	84	1.521 (1.043–2.218)	0.029 *	1.709 (1.124–2.598)	0.012 *
III	82	1.626 (1.117–2.366)	0.011 *	1.727 (1.139–2.620)	0.010 *
IV	34	3.648 (2.340–5.687)	0.000 *	2.637 (1.608–4.325)	< 0.001 *
Curative surgery	[297]				
No	123	Reference		Reference	
Yes	174	0.367 (0.276–0.488)	< 0.001 *	0.438 (0.312–0.616)	< 0.001 *
Jaundice					
No	136	Reference			
Yes	164	1.217 (0.917–1.615)	0.174		
Diabetes					
No	246	Reference			
Yes	54	1.016 (0.709–1.454)	0.933		
Hypertension					
No	201	Reference			
Yes	99	1.029 (0.762–1.389)	0.852		
Fatty liver					
No	278	Reference			
Yes	22	1.168 (0.689–1.980)	0.564		
Liver cirrhosis					
No	288	Reference			
Yes	12	1.619 (0.879–2.980)	0.122		
FBG (g/L)					
≤ 3.61	134	Reference		Reference	
> 3.61	166	1.918 (1.431–2.571)	< 0.001 *	1.478 (1.054–2.073)	0.023 *
PT (s)					
≤ 11.0	67	Reference		Reference	
> 11.0	233	1.587 (1.103–2.281)	0.013 *	1.251 (0.782–2.002)	0.350
PTA (%)					
≤ 94.0	177	Reference		Reference	
> 94.0	123	0.718 (0.538–0.968)	0.025 *	1.535 (0.921–2.558)	0.100
INR					
≤ 0.98	143	Reference		Reference	
> 0.98	157	1.585 (1.194–2.105)	0.001 *	1.879 (1.141–3.093)	0.013 *
APTT (s)					
≤ 26.5	154	Reference		Reference	
> 26.5	146	1.382 (1.042–1.832)	0.025 *	1.367 (0.212–8.820)	0.743
APTT-R					
≤ 0.98	153	Reference		Reference	
> 0.98	147	1.380 (1.041–1.831)	0.025 *	0.742 (0.113–4.856)	0.755
TT (s)					
≤ 16.9	45	Reference			
> 16.9	255	0.760 (0.520–1.110)	0.156		
PLT (×10^9^/L)					
≤ 301	239	Reference			
> 301	61	1.223 (0.874–1.711)	0.240		
CA19–9 (U/mL)	[290]				
≤ 37	83	Reference		Reference	
> 37	207	2.366 (1.654–3.386)	< 0.001 *	1.644 (1.089–2.482)	0.018 *
ALT (U/L)					
≤ 40	121	Reference			
> 40	179	1.076 (0.808–1.433)	0.615		
AST (U/L)					
≤ 40	132	Reference			
> 40	158	1.092 (0.820–1.455)	0.548		
GGT (U/L)	[293]				
≤ 40	55	Reference		Reference	
> 40	238	1.844 (1.219–2.791)	0.004 *	1.115 (0.673–1.848)	0.671
ALB (g/L)	[299]				
≤ 35	58	Reference			
> 35	241	0.794 (0.567–1.112)	0.179		

Asterisks indicated *P*-values of statistical significance. Continuous variables were shown as medians and ranges, and categorical variables were reported as numbers and percentages. For variables with missing data, the numbers of available cases were shown in square brackets

### Correlations of FBG and INR with clinicopathological characteristics

The correlations of FBG and INR with other clinicopathological characteristics are summarized in S1 Table and S2 Table.

In both the training and validation cohorts, a high FBG level was related to an elevated CA19-9 level (both *P* < 0.05) and non-curative surgery (both *P* < 0.05), both of which were independent prognostic factors for OS. High level of FBG was also connected in both cohorts to increased PLT (both *P* < 0.05), which has been widely investigated for its prognostic value in various malignancies although it was not significantly associated with OS in our study. In the training cohort, a high FBG level was also related to impaired liver function, including elevated ALT (*P* < 0.001), AST (*P* = 0.002), and GGT (*P* < 0.001) and the presence of jaundice (*P* < 0.001). Although not discovered in the validation cohort, elevated FBG indicated poorer short-term outcomes for patients with high FBG tended to suffer from more blood loss (400 vs 300 mL, *P* = 0.034) during surgery and longer hospital stays postoperatively (15 vs 13 days, *P* = 0.001).

Similarly, an elevated INR was related to poor tumor differentiation (both *P* < 0.05), prolonged PT (both *P* < 0.05), prolonged APTT (both *P* < 0.05), high APTT-R (both *P* < 0.05) and low PTA (both *P* < 0.05) in both cohorts. All these factors were adverse prognostic factors revealed by the univariate analysis. A high INR was also associated with decreased ALB, commonly regarded as an indicator of liver dysfunction and poor nutrition status, in both cohorts (both *P* < 0.05).

### Development and validation of a predictive nomogram for OS

**Fig. 3:**
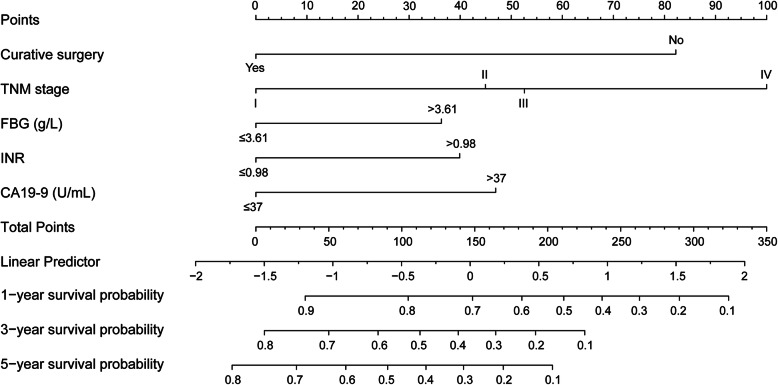
Nomogram integrating curative surgery, TNM stage, FBG, INR, and CA19-9 for 1-, 3-, and 5-year overall survival in patients with biliary tract cancer after surgery

**Fig. 4:**
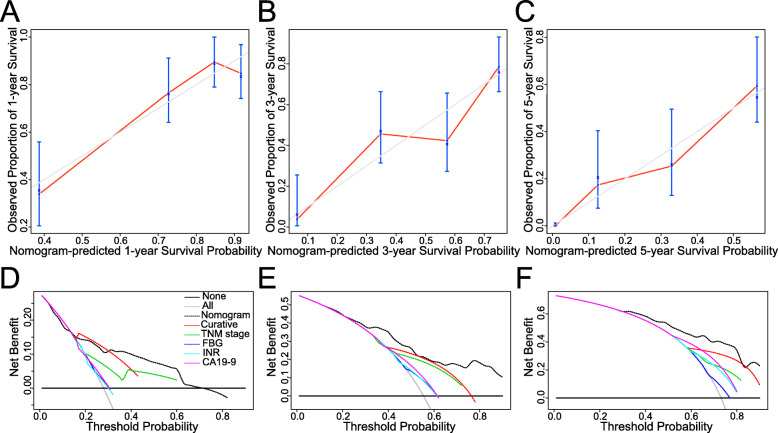
Calibration curves and decisive curve analysis of the nomogram in the validation cohort. **a-c** 1-, 3- and 5-year calibration curves. **d-f** 1-, 3- and 5-year decisive curve analyses

A nomogram for 1-, 3-, and 5-year OS prediction was constructed based on Cox regression analyses in the training cohort, integrating curative surgery, TNM stage, FBG, INR, and CA19-9 (Fig. 3). The C index of the nomogram was 0.729 (95% CI: 0.691–0.767) in the training cohort and 0.731 (95% CI: 0.674–0.788) in the validation cohort, which was superior to the ones of individual prognostic factors, including curative surgery (0.597, training cohort; 0.649, validation cohort), TNM stage (0.612, training cohort; 0.655, validation cohort), FBG (0.571, training cohort; 0.545, validation cohort), INR (0.562, training cohort; 0.549, validation cohort), and CA19-9 (0.589, training cohort; 0.560, validation cohort). Calibration curves for the 1-, 3-, and 5-year survival probability after surgery displayed considerable agreement between the nomogram-predicted OS and the observed survival in the training cohort (S1 Fig. A-C) and the validation cohort (Fig. 4a-c). DCA was conducted to assess the net benefit of the nomogram in clinical applications. The nomogram generated the most net benefit across a broad range of threshold probability for 1-, 3-, and 5-year OS in both the training cohort (S1 Fig. D-F) and the validation cohort (Fig. 4d-f), suggesting that the nomogram outperformed TNM staging and the other four independent prognostic factors.

To addressed the issue whether the inclusion of different types of malignant biliary neoplasms could potentially impact on the predictive performance of the nomogram, the nomogram was also validated in ICC, ECC, and GBC, respectively. As displayed by the calibration curves, the predicted survival probability was in good agreement with the observed survival in ICC (S2 Fig. A), ECC (S2 Fig. B), and GBC (S2 Fig. C), respectively, suggesting that the nomogram had satisfactory consistency in different types of biliary tract cancer. Time-dependent AUC curves indicated that the discrimination of the nomogram in ICC (S3 Fig. A), ECC (S3 Fig. B) and GBC (S3 Fig. C) was obviously superior to TNM staging. DCA were also performed, indicating that the nomogram generated more net benefit than TNM staging, whether in ICC (S4 Fig. A-C), ECC (S4 Fig. D-F) or GBC (S4 Fig. G-I).

### A novel stratification model based on the nomogram

**Fig. 5:**
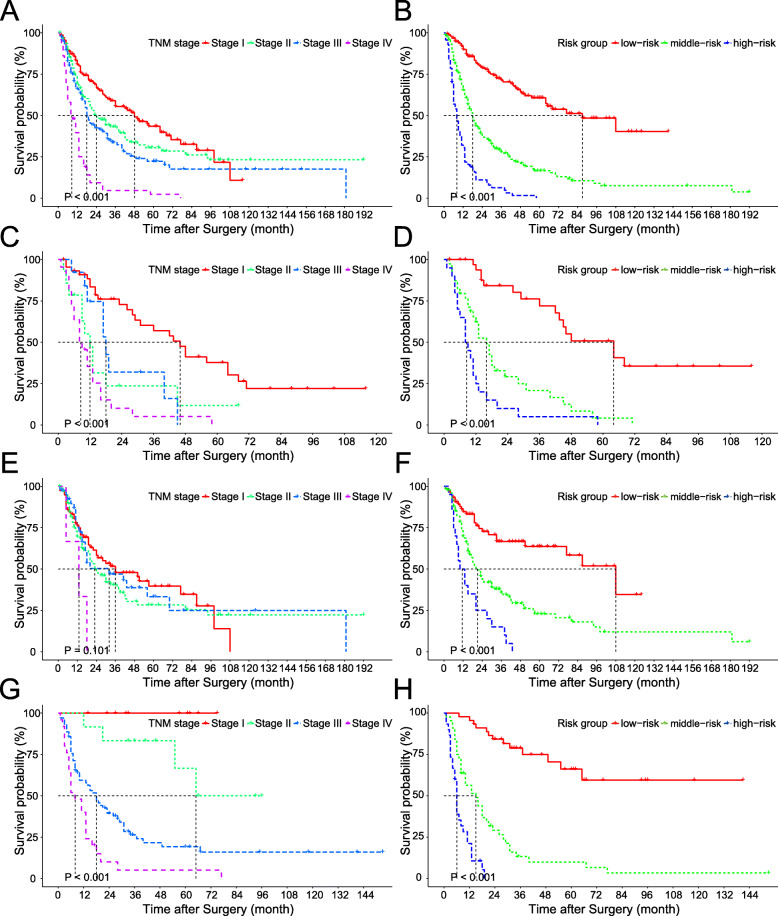
Kaplan-Meier survival curves according to TNM staging and the novel stratification model. **a-b** Survival curves of all patients without missing data. **c-d** Survival curves of patients with ICC. **e-f** Survival curves of patients with ECC. **g-h** Survival curves of patients with GBC

According to the 7th edition of the AJCC TNM staging system, the median OS of patients at stage 0-I, II, III, and IV was 48, 24, 18, and 8 months, respectively (*P* < 0.001, Fig. 5a). Based on the total points of each patient derived from the nomogram, we generated a novel stratification model. All patients without missing data in any individual prognostic factor of the nomogram were subsequently classified into 3 groups using X-tile software: low-risk (*n* = 156, score: 0–126.9), middle-risk (*n* = 209, score: 127.0–221.9), and high-risk (*n* = 70, score: 222.0–305.5). The median OS was 87, 18, and 8 months for the low-risk, middle-risk, and high-risk groups, respectively (*P* < 0.001, Fig. 5b). The C index of the novel stratification model was 0.702 compared to 0.626 for the TNM staging (*P* < 0.001), indicating that the novel stratification model had a better performance than TNM staging in survival prediction.

As the AJCC TNM staging system differs for ICC, ECC and, GBC, the novel stratification model was compared with TNM staging in different subgroups according to the type of biliary tract cancer. Kaplan-Meier survival curves showed that TNM staging failed to distinguish the prognoses of patients with ICC (Fig. 5c) and ECC (Fig. 5e) well, but was satisfactory in patients with GBC (Fig. 5g). The prognoses, however, were quite separated according to the novel stratification model in ICC (Fig. 5d), ECC (Fig. 5f), and GBC (Fig. 5h), respectively. The C index also revealed that the ability to predict survival by the novel stratification model was superior to that of TNM staging in ICC (0.730 vs 0.699, *P* < 0.001), ECC (0.628 vs 0.537, *P* < 0.001), and GBC (0.767 vs 0.694, *P* < 0.001). We also investigated the performance of TNM staging and the novel stratification model in patients who underwent curative and non-curative surgeries, respectively. Kaplan-Meier survival curves suggested that the novel stratification model was superior to TNM staging in distinguishing the prognoses of patients who underwent curative (S2 Fig. A-B) and non-curative surgery (S2 Fig. C-D), which was also indicated by the C index (0.652 vs 0.606 for curative surgery, *P* < 0.001; 0.624 vs 0.618 for non-curative surgery, *P* = 0.225) although statistical significance was not obtained in patients who underwent non-curative surgery.

## Discussion

In this study, we retrospectively investigated the prognostic significance of preoperative coagulation indices, including FBG, PT, PTA, INR, APTT, APTT-R, TT, and PLT, in patients with BTC who underwent surgery. High FBG, PT, INR, APTT, and APTT-R levels, and decreased PTA and TT levels indicated poorer OS, while PLT was not significantly associated with OS. FBG (HR = 1.478, *P* = 0.023) and INR (HR = 1.879, *P* = 0.013), along with curative surgery, TNM staging, and CA19–9, were identified as independent prognostic factors for BTC patients receiving surgery. Notably, they had the best predictive accuracy among the coagulation indices considered. In addition, FBG and INR were related to other clinicopathological characteristics that indicated more serious medical conditions or poorer prognoses.

FBG, also known as Factor I, is a multifunction glycoprotein synthesized by hepatocytes. It can be also derived from tumor cells in patients with cancer. Studies have found that FBG makes considerable contributes to tumorigenesis, progression, and hematogenous metastasis. As a common protein component of extracellular matrix, FBG facilitates the formation of tumor stroma by providing structure [19] and enhancing the chemotactic properties of stromal cells [20]. Serving as a scaffold, FBG binds to growth factors like VEGF [21] and FGF-2 [22], which provide growth signals for angiogenesis of tumors. FBG also promotes the adhesion of circulating cancer cells to the endothelium of vascular vessels, leading to increased risk of cancer metastasis [23]. An in vitro study concluded that FBG promoted cancer cell migration and invasion by inducing epithelial-mesenchymal transition [24]. Studies have confirmed that a high FBG level is an unfavorable prognostic factor for different malignant tumors [16, 17, 24–26], which is in accordance with the results of the present study. Patients with high FBG level also tended to have a poorer short-term clinical outcome, as revealed in the training cohort. In addition, this study found that high FBG was related to non-curative surgery and elevated CA19–9, both of which were independent prognostic factors for patients with BTC receiving surgery. Although associated with an elevated PLT, high FBG was not significantly correlated with abnormalities of other coagulation indices. Interestingly, as the training cohort suggested, high FBG was also related to liver dysfunction, which reflected the disease status of BTC to a certain extent.

INR is a standardized index to measure the clotting tendency based on the ratio of an individual’s PT and the normal mean PT. INR and PT are important parameters to evaluate activity of the extrinsic pathway of coagulation. Increased INR or prolonged PT indicate a compromised extrinsic clotting system. Cancer-associated activation of the coagulation system leads to a subsequent deficiency of coagulation factors, resulting in PT prolongation. The decreased capacity of liver biosynthesis in cancer patients may also have a role to play in this situation [16]. Previous research has shown that prolonged PT is associated with more dismal prognoses for a variety of malignancies, including lung cancer [18] and hepatocellular carcinoma [27]. We confirmed that INR was independently associated with OS in patients with BTC, which is consistent with an earlier study [28]. We also found that elevated INR was correlated with poor tumor differentiation, decreased level of ALB, and abnormalities in other coagulation indices, including PT, PTA, APTT, and APTT-R.

Overexpression of procoagulant proteins is one of the major reasons why patients with malignant tumors tend to suffer from aberrantly activated coagulation [10, 11]. Tumor-cell derived tissue factor (TF, also known as Factor III) contributes to the coagulopathy in cancer to a great extent [10]. By binding with Factor VIIa, TF trigger coagulation cascade by activating Factor X, and thus plays an essential role in the extrinsic pathway of the coagulation system. The extrinsic pathway is probably more perturbed than the intrinsic pathway. Therefore, it is reasonable that INR, an indicator of the extrinsic pathway based on PT and PTA, was more closely correlated with the prognosis of BTC than indicators of the intrinsic pathway (APTT and APTT-R). Fibrinolysis system is also involved in the disorder of hemostatic system in cancer patients. Although it is common that urokinase-type plasminogen activator, tissue-type plasminogen activator as well as their receptor were activated during tumor growth, activation of the fibrinolysis inhibitors is also observed [12]. This kind of contradictory situation might lead the result that TT, an indicator of fibrinolysis system, failed to be independent prognostic factor for BTC. It has been revealed that platelets participate in the coagulopathy and progression of cancer [9]. The result that PLT was not associated with OS in our study suggests that the function of platelets possibly matters more than the count.

Previously, Wang et al. showed that a prolonged PT was related to a high recurrence rate and poor prognosis for patients with ICC following curative resection [29]. A retrospective and in vitro study with a relatively small sample size (*n* = 115) conducted by Shu et al. showed the clinical significance of elevated preoperative FBG in GBC patients after surgical resection [24]. Although aberrant coagulation system has been suggested to negatively impact on patients with ICC or GBC, evidence regarding the prognosis value of coagulation indices in BTC still lacks. Besides, few prognostic models based on coagulation indices have been built for BTC so far. Therefore, thorough and comprehensive investigations of the prognostic value of coagulation indices and their relationships with other clinical characteristics in BTC are still needed.

Despite the wide application of the AJCC TNM staging system to evaluate the clinical outcome in patients with BTC, it has been recognized that the prognoses are varied in patients within the same TNM stage. Other pathological characteristics such as tumor subtype and tumor differentiation are also utilized in the survival estimation of BTC patients. A consensus, however, has been reached that not only tumor intrinsic properties but also patient-related factors impact the prognoses of patients with cancer [30]. Given that coagulation factors, especially FBG and INR, were revealed to be associated with overall survival of BTC patients in our study, a nomogram integrating the five independent prognostic factors revealed by multivariate analysis was developed. The nomogram took tumor-related, patient-related, and treatment-related factors into comprehensive account and was shown to have considerable performance in the aspect of discrimination, consistency, and clinical net benefit. Based on the total points of the nomogram, we also constructed a novel stratification model, which classified the patients into low-risk, middle-risk, and high-risk groups. The prognoses of patients who received surgery for biliary tract cancer within different risk groups were well-separated and the predictive accuracy of the novel stratification model outperformed that of TNM staging, no matter which type of tumor and whether the surgery was curative.

Meanwhile, there were some limitations that should be addressed in our study. Firstly, this was a retrospective study conducted at a single center in China; thus, selection bias might have been inevitable. Whether the cut-off values of the coagulation indices are optimal for other areas of the world and ethnicities remains to be confirmed. Secondly, although the nomogram was validated in both the training and validation cohorts, it still lacks external validation by data from other medical centers. Besides, the inclusion of different types of biliary tract cancer could amplify the heterogeneity of patients. Although rigorous subgroups analyses have been conducted to shown that the reliability of the results was not obviously compromised by such heterogeneity, researches are needed to confirm the results further in ICC, ECC, and GBC, respectively. In addition, the missing data in our study might have detrimental effects on the accuracy of the statistical analyses. Yet, these influences were likely slight due to a relatively large sample size. Last but not least, progression-free survival, which was another important aspect of the prognosis, and adjuvant therapy, which might play a potential role in the survival of patients with BTC, were not included in the current study due to limited follow-up data. Taking these limitations into consideration, large-scale, multi-center, and prospective studies are required to verify our conclusions in BTC patients of different ethnicities.

## Conclusions

In conclusion, coagulation indices are valuable for predicting the prognosis of patients with BTC who underwent surgical resection, with FBG and INR being independent prognostic factors for OS. The nomogram integrating FBG, INR, curative surgery, and CA19–9 was established and verified to predict survival for BTC patients after surgery. The novel stratification model based on the nomogram better distinguished the prognoses of BTC patients than TNM staging.

## Supplementary Information

**Additional file 1: S1 Table.** Correlations between FBG and Other Clinicopathological Characteristics.

**Additional file 2: S2 Table.** Correlations between INR and Other Clinicopathological Characteristics.

**Additional file 3: S1 Fig**. Calibration curves and decisive curve analysis of the nomogram in the training cohort. A-C: 1-, 3- and 5-year calibration curves. D-F: 1-, 3- and 5-year decisive curve analyses.

**Additional file 4: S2 Fig**. Calibration curves in ICC (A), ECC (B), and GBC (C). Red: 1-year calibration curves; blue: 3-year calibration curves; green: 5-year calibration curves.

**Additional file 5: S3 Fig**. Time-dependent area under receiver operating characteristic curves in ICC (A), ECC (B), and GBC (C).

**Additional file 6: S4 Fig**. Decisive curve analysis in ICC (A-C), ECC (D-F), and GBC (G-I).

**Additional file 7: S5 Fig**. Kaplan-Meier survival curves of patients receiving curative and non-curative surgery, respectively. A-B: survival curves of patients who underwent curative surgery. C-D: survival curves of patients who underwent non-curative surgery.

## Data Availability

The datasets used and analyzed during the present study were retrospectively collected from the medical records of Peking Union Medical College Hospital. Permissions have been granted by the Institutional Review Board of Peking Union Medical College Hospital to access the original medical records for the present study. All data generated or analyzed during this study are included in this paper and the supplementary materials.

## Abbreviations

AJCC: American Joint Committee on Cancer
ALB: Albumin
ALT: Alanine aminotransferase
APTT: Activated partial thromboplastin time
APTT-R: Activated partial thromboplastin time ratio
AST: Aspartate aminotransferase
BTC: Biliary tract cancer
CA19-9: Carbohydrate antigen 19-9
C index: Harrell’s concordance index
DCA: Decisive curve analysis
ECC: Extrahepatic cholangiocarcinoma
FBG: Fibrinogen
GBC: Gallbladder cancer
GGT: γ-glutamyl transpeptidase
ICC: Intrahepatic cholangiocarcinoma
INR: International normalized ratio
OS: Overall survival
PLT: Platelet count
PT: Prothrombin time
PTA: Prothrombin time activity
AUC: Area under receiver operating characteristic
TNM: Tumor-node-metastasis
TT: Thrombin time
