# Supplementary material for: Comprehensive analysis of coagulation indices for predicting survival in patients with biliary tract cancer
Source: BMC Cancer. 2021 Aug 25;21:953. doi: 10.1186/s12885-021-08684-w (PMC8390227; doi:10.1186/s12885-021-08684-w)
Supplement: Supplementary file 2 — Additional file 2: S2 Table. Correlations between INR and Other Clinicopathological Characteristics. [file 12885_2021_8684_MOESM2_ESM.docx]

**S2 Table. Correlations between INR and Other Clinicopathological Characteristics**

| Characteristics | Training cohort (n=300) | | | Validation cohort (n=150) | | |
| --- | --- | --- | --- | --- | --- | --- |
|  | **INR≤0.98 (n=143)** | **INR>0.98 (n=157)** | **P** | **INR≤0.98 (n=57)** | **INR>0.98 (n=93)** | ***P*** |
| Age (year) |  |  | 0.443 |  |  | 0.565 |
| ≤60 | 71 (49.7%) | 71 (45.2%) |  | 20 (35.1%) | 37 (39.8%) |  |
| >60 | 72 (50.3%) | 86 (54.8%) |  | 37 (64.9%) | 56 (60.2%) |  |
| Sex |  |  | 0.863 |  |  | 0.792 |
| Female | 67 (46.9%) | 72 (45.9%) |  | 27 (47.4%) | 42 (45.2%) |  |
| Male | 76 (53.1%) | 85 (54.1%) |  | 30 (52.6%) | 51 (54.8%) |  |
| Malignancy type |  |  | 0.105 |  |  | 0.216 |
| ICC | 38 (26.6%) | 27 (17.2%) |  | 12 (21.1%) | 22 (23.7%) |  |
| ECC | 65 (45.5%) | 87 (55.4%) |  | 24 (42.1%) | 49 (52.7%) |  |
| GBC | 40 (27.9%) | 43 (27.4%) |  | 21 (36.8%) | 22 (23.4%) |  |
| Tumor Differentiation | [138] | [148] | 0.025 * | [54] | [85] | 0.017 * |
| Poor | 42 (30.4%) | 64 (43.2%) |  | 15 (27.8%) | 41 (48.2%) |  |
| Modest-well | 96 (69.6%) | 84 (56.8%) |  | 39 (72.2%) | 44 (51.8%) |  |
| Tumor size (cm) | [137] | [145] | 0.850 | [55] | [90] | 0.688 |
| ≤2 | 62 (45.3%) | 64 (44.1%) |  | 25 (45.5%) | 44 (48.9%) |  |
| >2 | 75 (54.7%) | 81 (55.9%) |  | 30 (54.5%) | 46 (51.1%) |  |
| TNM stage |  |  | 0.728 |  |  | 0.422 |
| I | 49 (34.3%) | 51 (32.5%) |  | 17 (29.8%) | 24 (25.8%) |  |
| II | 41 (28.7%) | 43 (27.4%) |  | 16 (28.1%) | 31 (33.3%) |  |
| III | 35 (24.5%) | 47 (29.9%) |  | 20 (35.1%) | 25 (26.9%) |  |
| IV | 18 (12.5%) | 16 (10.2%) |  | 4 (7.0%) | 13 (14.0%) |  |
| Curative surgery |  | [154] | 0.855 |  |  | 0.621 |
| No | 60 (42.0%) | 63 (40.9%) |  | 24 (42.1%) | 43 (46.2%) |  |
| Yes | 83 (58.0%) | 91 (59.1%) |  | 33 (57.9%) | 50 (53.8%) |  |
| Jaundice |  |  | 0.333 |  |  | 0.333 |
| No | 69 (48.3%) | 67 (42.7%) |  | 31 (54.4%) | 43 (46.2%) |  |
| Yes | 74 (51.7%) | 90 (57.3%) |  | 26 (45.6%) | 50 (53.8%) |  |
| Diabetes |  |  | 0.327 |  |  | 0.403 |
| No | 114 (79.7%) | 132 (84.1%) |  | 42 (73.7%) | 74 (79.6%) |  |
| Yes | 29 (20.3%) | 25 (15.9%) |  | 15 (26.3%) | 19 (20.4%) |  |
| Hypertension |  |  | 0.055 |  |  | 0.981 |
| No | 88 (61.5%) | 113 (72.0%) |  | 43 (75.4%) | 70 (75.3%) |  |
| Yes | 55 (38.5%) | 44 (28.0%) |  | 14 (24.6%) | 23 (24.7%) |  |
| Fatty liver |  |  | 0.820 |  |  | 0.030 * |
| No | 132 (92.3%) | 146 (93.0%) |  | 52 (91.2%) | 92 (98.9%) |  |
| Yes | 11 (7.7%) | 11 (7.0%) |  | 5 (8.8%) | 1 (1.1%) |  |
| Liver cirrhosis |  |  | 0.671 |  |  | >0.999 |
| No | 138 (96.5%) | 150 (95.5%) |  | 56 (98.2%) | 91 (97.8%) |  |
| Yes | 5 (3.5%) | 7 (4.5%) |  | 1 (1.8%) | 2 (2.2%) |  |
| FBG (g/L) |  |  | 0.098 |  |  | 0.568 |
| ≤3.61 | 71 (49.7%) | 63 (40.1%) |  | 26 (45.6%) | 38 (40.9%) |  |
| >3.61 | 72 (50.3%) | 94 (59.9%) |  | 31 (54.4%) | 55 (59.1%) |  |
| PT (s) |  |  | <0.001 * |  |  | <0.001 * |
| ≤11.0 | 63 (44.1%) | 4 (2.5%) |  | 27 (47.4%) | 5 (5.4%) |  |
| >11.0 | 80 (55.9%) | 153 (97.5%) |  | 30 (52.6%) | 88 (94.6%) |  |
| PTA (%) |  |  | <0.001 * |  |  | <0.001 * |
| ≤94.0 | 30 (21.0%) | 147 (93.6%) |  | 12 (21.1%) | 87 (93.5%) |  |
| >94.0 | 113 (79.0%) | 10 (6.4%) |  | 45 (78.9%) | 6 (6.5%) |  |
| APTT (s) |  |  | <0.001 * |  |  | <0.001 * |
| ≤26.5 | 101 (70.6%) | 53 (33.8%) |  | 43 (75.4%) | 34 (36.6%) |  |
| >26.5 | 42 (29.4%) | 104 (66.2%) |  | 14 (24.6%) | 59 (63.4%) |  |
| APTT-R |  |  | <0.001 * |  |  | <0.001 * |
| ≤0.98 | 101 (70.6%) | 52 (33.1%) |  | 43 (75.4%) | 34 (36.6%) |  |
| >0.98 | 42 (29.4%) | 105 (66.9%) |  | 14 (24.6%) | 59 (63.4%) |  |
| TT (s) |  |  | 0.150 |  |  | 0.023 * |
| ≤16.9 | 17 (11.9%) | 28 (17.8%) |  | 6 (10.5%) | 24 (25.8%) |  |
| >16.9 | 126 (88.1%) | 129 (82.2%) |  | 51 (89.5%) | 69 (74.2%) |  |
| PLT (×10^9^/L) |  |  | 0.982 |  |  | 0.085 |
| ≤301 | 114 (79.7%) | 125 (79.6%) |  | 51 (89.5%) | 73 (78.5%) |  |
| >301 | 29 (20.3%) | 32 (20.4%) |  | 6 (10.5%) | 20 (21.5%) |  |
| CA19-9 (U/mL) | [138] | [152] | 0.091 | [56] | [92] | 0.089 |
| ≤37 | 46 (33.3%) | 37 (24.3%) |  | 20 (35.7%) | 21 (22.8%) |  |
| >37 | 92 (66.7%) | 115 (75.7%) |  | 36 (64.3%) | 71 (77.2%) |  |
| ALT (U/L) |  |  | 0.584 |  |  | 0.047 * |
| ≤40 | 60 (42.0%) | 61 (38.9%) |  | 27 (47.4%) | 29 (31.2%) |  |
| >40 | 83 (58.0%) | 96 (61.1%) |  | 30 (52.6%) | 64 (68.8%) |  |
| AST (U/L) | [141] | [149] | 0.255 | [52] | [87] | 0.037 |
| ≤40 | 69 (48.9%) | 63 (42.3%) |  | 26 (50.0%) | 28 (32.2%) |  |
| >40 | 72 (51.1%) | 86 (57.7%) |  | 26 (50.0%) | 59 (67.8%) |  |
| GGT (U/L) | [141] | [152] | 0.053 | [52] | [87] | 0.084 |
| ≤40 | 33 (23.4%) | 22 (14.5%) |  | 14 (26.9%) | 13 (14.9%) |  |
| >40 | 108 (76.6%) | 130 (85.5%) |  | 38 (73.1%) | 74 (85.1%) |  |
| ALB (g/L) | [142] |  | <0.000 * | [56] |  | 0.001 * |
| ≤35 | 15 (10.6%) | 43 (27.4%) |  | 4 (7.1%) | 27 (29.0%) |  |
| >35 | 127 (89.4%) | 114 (72.6%) |  | 52 (92.9%) | 66 (71.0%) |  |
| Blood loss during surgery (mL) | 300 (0–2 600) | 300 (0–8 000) | 0.393 | 300 (0–2 000) | 300 (10–4 000) | 0.746 |
| Postoperative hospitalization (day) | 14 (1–154) | 14 (2–132) | 0.615 | 12 (2–91) | 12 (3–55) | 0.490 |
| Postoperative complication |  |  | 0.147 |  |  | 0.586 |
| No | 98 (68.5%) | 95 (60.5%) |  | 33 (57.9%) | 58 (62.4%) |  |
| Yes | 45 (31.5%) | 62 (39.5%) |  | 24 (42.1%) | 35 (37.6%) |  |

Asterisks indicated *P*-values of statistical significance. Continuous variables were shown as medians and ranges, and categorical variables were reported as numbers and percentages. For variables with missing data, the numbers of available cases were shown in square bracket.
