# Supplementary material for: Comprehensive analysis of coagulation indices for predicting survival in patients with biliary tract cancer
Source: BMC Cancer. 2021 Aug 25;21:953. doi: 10.1186/s12885-021-08684-w (PMC8390227; doi:10.1186/s12885-021-08684-w)
Supplement: Supplementary file 3 — Additional file 3: S1 Fig. Calibration curves and decisive curve analysis of the nomogram in the training cohort. A-C: 1-, 3- and 5-year calibration curves. D-F: 1-, 3- and 5-year decisive curve analyses. [file 12885_2021_8684_MOESM3_ESM.docx]

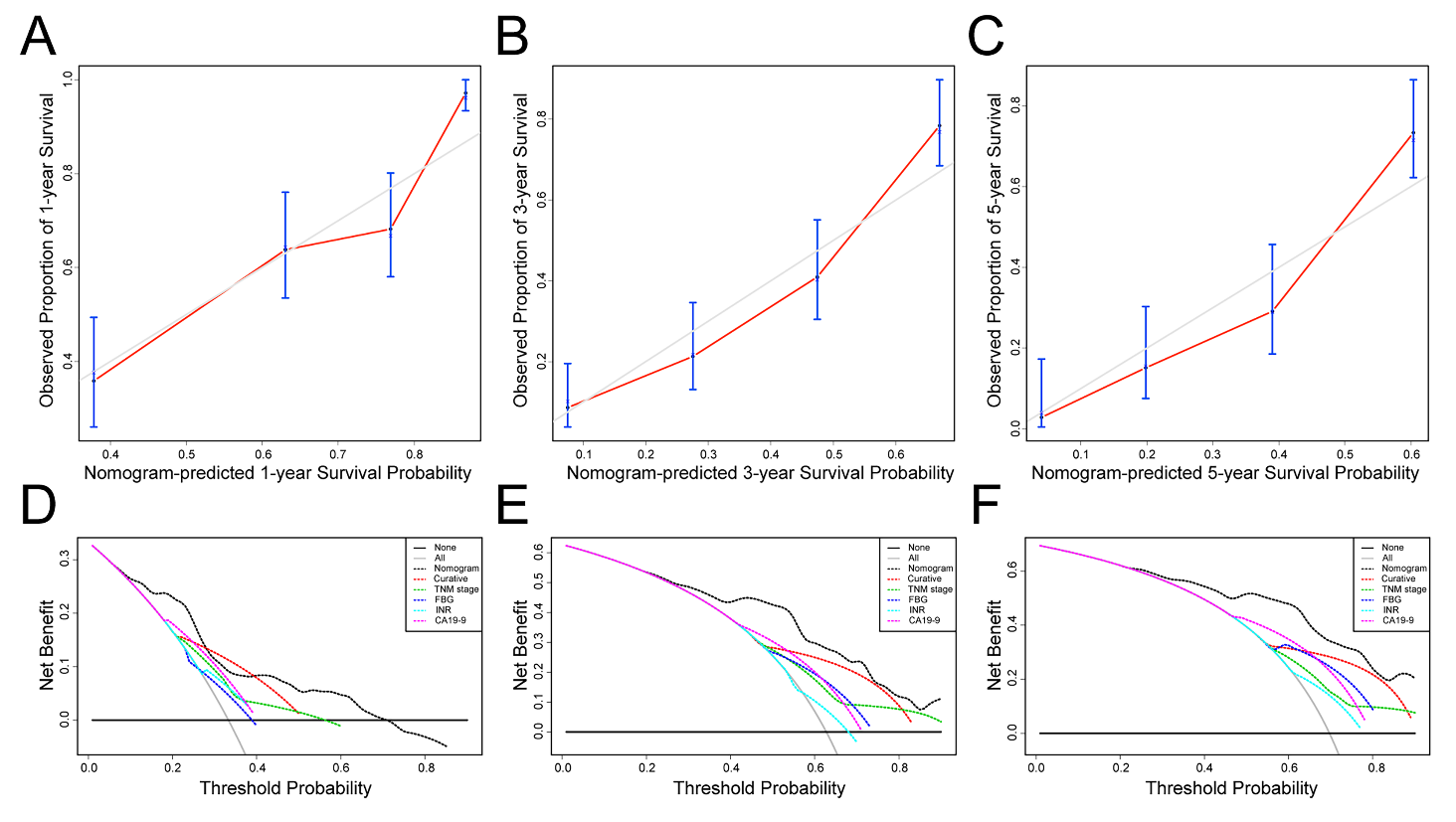


**S1 Fig. Calibration curves and decisive curve analysis of the nomogram in the training cohort.** A-C: 1-, 3- and 5-year calibration curves. D-F: 1-, 3- and 5-year decisive curve analyses.
