# Supplementary material for: Comprehensive analysis of coagulation indices for predicting survival in patients with biliary tract cancer
Source: BMC Cancer. 2021 Aug 25;21:953. doi: 10.1186/s12885-021-08684-w (PMC8390227; doi:10.1186/s12885-021-08684-w)
Supplement: Supplementary file 4 — Additional file 4: S2 Fig. Calibration curves in ICC (A), ECC (B), and GBC (C). Red: 1-year calibration curves; blue: 3-year calibration curves; green: 5-year calibration curves. [file 12885_2021_8684_MOESM4_ESM.docx]

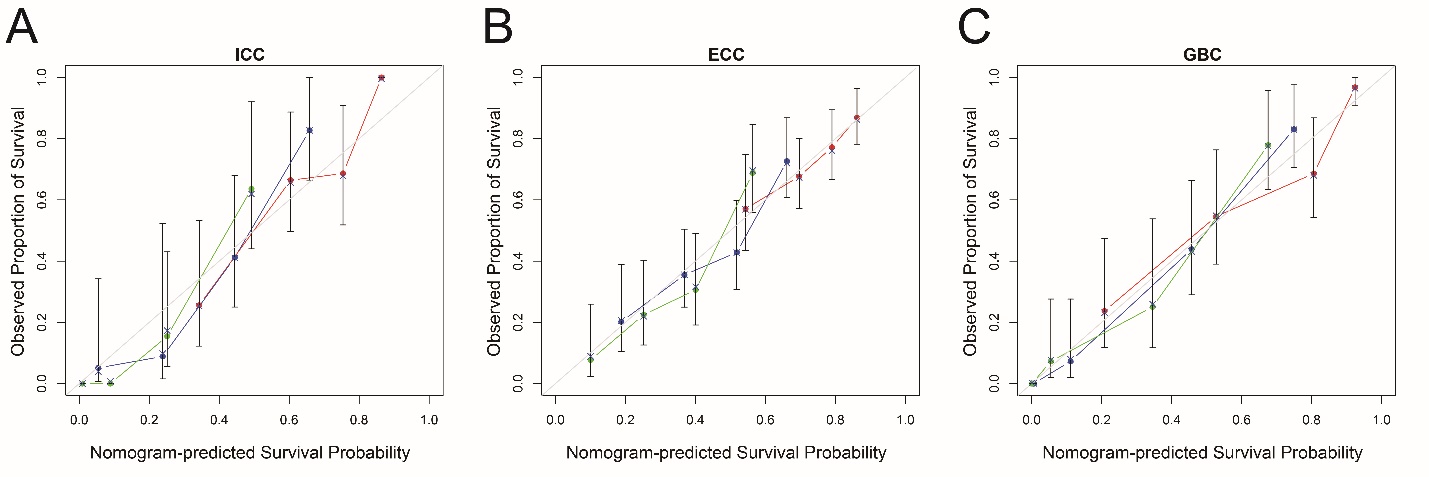


**S2 Fig. Calibration curves in ICC (A), ECC (B), and GBC (C).** Red: 1-year calibration curves; blue: 3-year calibration curves; green: 5-year calibration curves.
