# Supplementary material for: Comprehensive analysis of coagulation indices for predicting survival in patients with biliary tract cancer
Source: BMC Cancer. 2021 Aug 25;21:953. doi: 10.1186/s12885-021-08684-w (PMC8390227; doi:10.1186/s12885-021-08684-w)
Supplement: Supplementary file 5 — Additional file 5: S3 Fig. Time-dependent area under receiver operating characteristic curves in ICC (A), ECC (B), and GBC (C). [file 12885_2021_8684_MOESM5_ESM.docx]

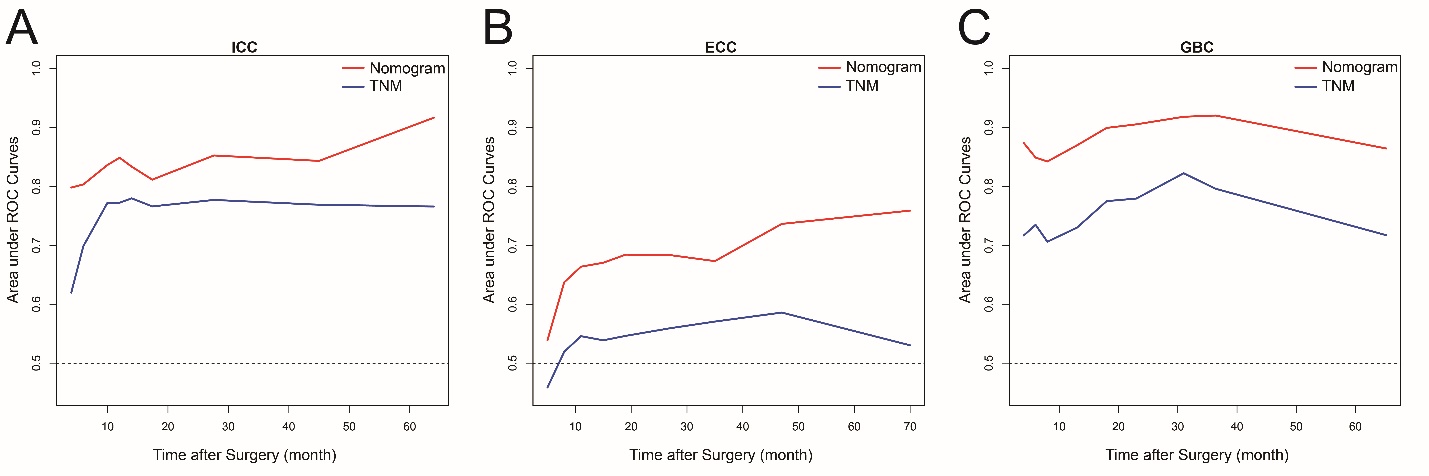


**S3 Fig. Time-dependent area under receiver operating characteristic curves in ICC (A), ECC (B), and GBC (C).**
