# Supplementary figures and images for: Comprehensive analysis of coagulation indices for predicting survival in patients with biliary tract cancer
Source: BMC Cancer. 2021 Aug 25;21:953. doi: 10.1186/s12885-021-08684-w (PMC8390227; doi:10.1186/s12885-021-08684-w)

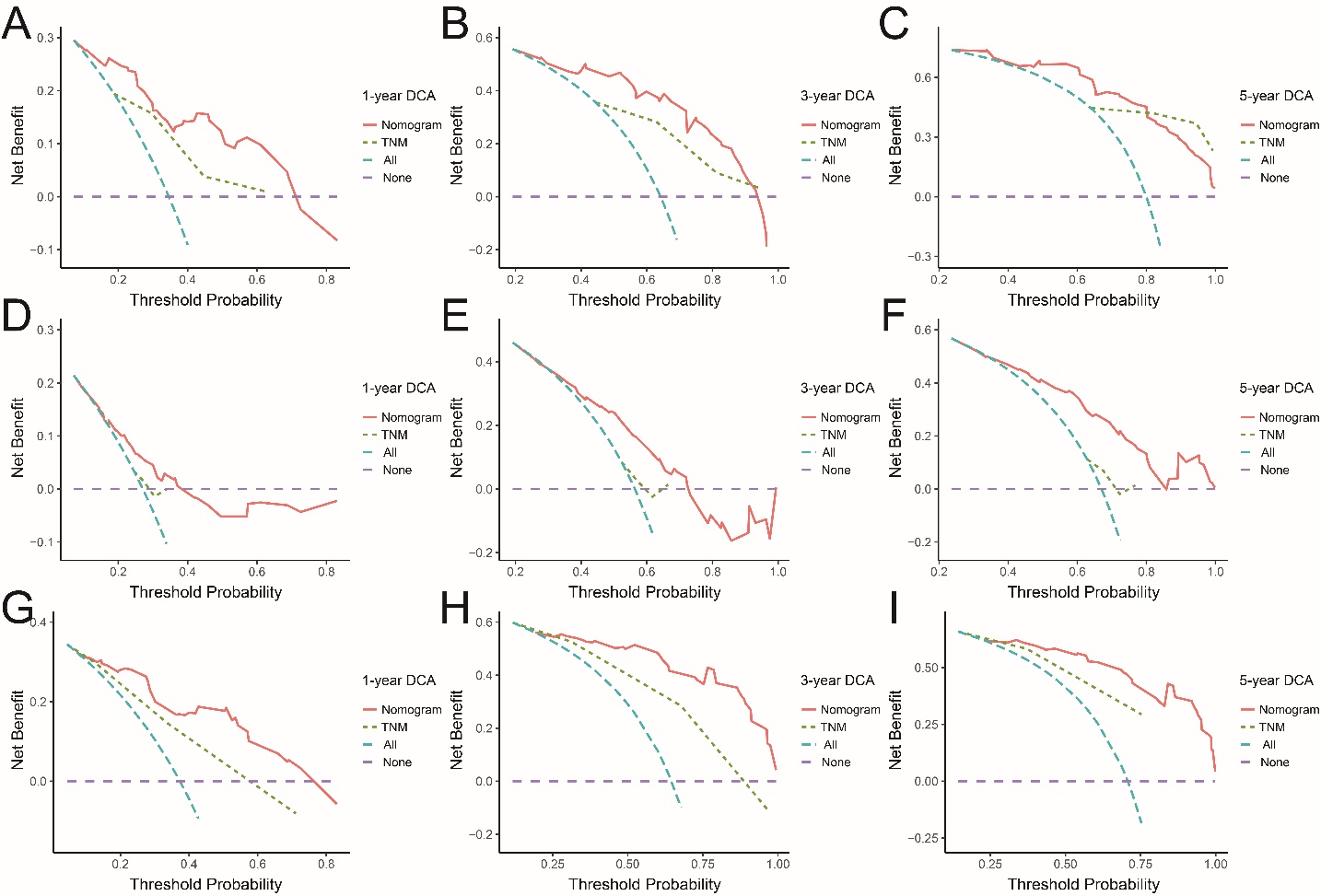


**S4 Fig. Decisive curve analysis in ICC (A-C), ECC (D-F), and GBC (G-I).**

Supplement: Supplementary file 6 — Additional file 6: S4 Fig. Decisive curve analysis in ICC (A-C), ECC (D-F), and GBC (G-I). [file 12885_2021_8684_MOESM6_ESM.docx]
