# Supplementary material for: Comprehensive analysis of coagulation indices for predicting survival in patients with biliary tract cancer
Source: BMC Cancer. 2021 Aug 25;21:953. doi: 10.1186/s12885-021-08684-w (PMC8390227; doi:10.1186/s12885-021-08684-w)
Supplement: Supplementary file 7 — Additional file 7: S5 Fig. Kaplan-Meier survival curves of patients receiving curative and non-curative surgery, respectively. A-B: survival curves of patients who underwent curative surgery. C-D: survival curves of patients who underwent non-curative surgery. [file 12885_2021_8684_MOESM7_ESM.docx]

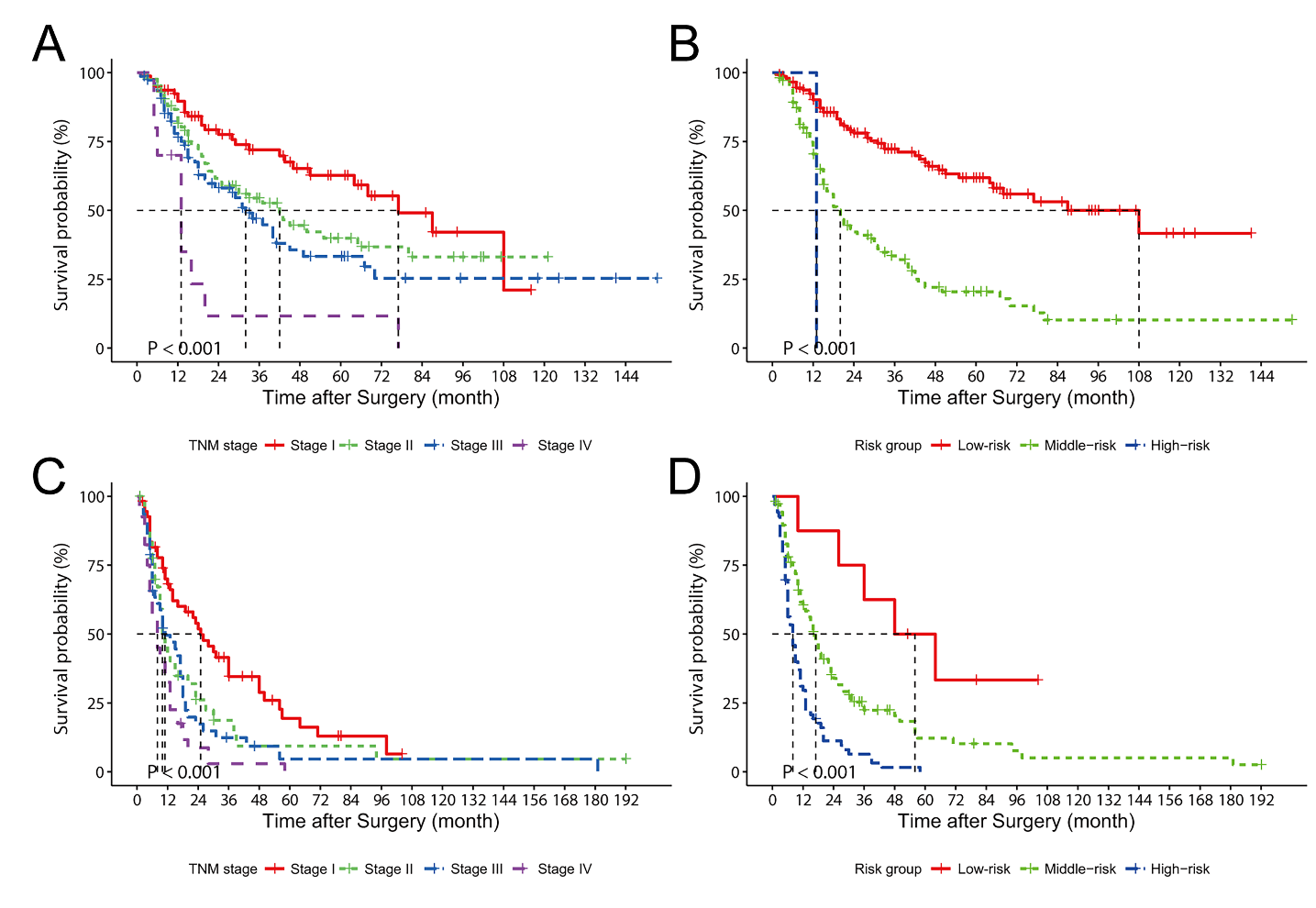


**S5 Fig. Kaplan-Meier survival curves of patients receiving curative and non-curative surgery, respectively.** A-B: survival curves of patients who underwent curative surgery. C-D: survival curves of patients who underwent non-curative surgery.
